# Effect of Aromatic Petroleum Resin on Damping Properties of Polybutyl Methacrylate

**DOI:** 10.3390/polym12030543

**Published:** 2020-03-02

**Authors:** Songhan Wan, Saisai Zhou, Xing Huang, Songbo Chen, Shuwei Cai, Xianru He, Rui Zhang

**Affiliations:** 1 School of Materials Science and Engineering, Southwest Petroleum University, Chengdu 610500, China; songhanwan@foxmail.com (S.W.); zhousaisai@xinpoint.com (S.Z.); huangxing0226@foxmail.com (X.H.); chen756424838@foxmail.com (S.C.); victor8951@foxmail.com (S.C.); 2 Institute für Physik, Universität Rostock, Albert-Einstein-Str. 23-24, 18051 Rostock, Germany; rui.zhang@uni-rosotck.de

**Keywords:** polybutyl methacrylate (PBMA), aromatic petroleum resin (C9), styrene, damping properties

## Abstract

The damping properties of polybutyl methacrylate (PBMA)/aromatic petroleum resin (C9) composite were investigated in this work. In particular, a trace of styrene (St) was introduced to copolymerize with PBMA to improve the compatibility between C9 and matrix. The structure of the copolymer, P(BMA-co-St), was characterized by FTIR and ^1^HNMR. The P(BMA-co-St)/C9 composites were tested by differencial scanning calorimetry (DSC), scanning electron microscopy (SEM) and dynamical mechanical analysis (DMA). DSC curves of all P(BMA-co-1wt%St)/C9 composites expressed only one glass transition temperature (*Tg*). SEM images showed that C9 had good compatibility with the matrix after St was introduced. DMA curves indicated that the addition of C9 had a positive effect on the damping properties of PBMA. The loss tangent (tanδ) peak moved to a higher temperature with the increment content of C9, and the effective damping temperature range increased significantly. The influence of aromatic resin C9 and aliphatic resin (C5) on PBMA damping performance was compared. It was further shown that C9 with benzene ring effectively improved the damping performance of PBMA.

## 1. Introduction

The vibration and noise pollution not only affect daily live seriously, but also decrease the stability and accuracy of equipment operation. In order to reduce vibration and noise, more and more attention has been paid to the design and preparation of damping materials [1,2,3]. Polymer materials are widely used in the field of damping materials because of the unique viscoelasticity in the glass transition zone, which can convert mechanical energy into internal energy dissipation [4,5,6,7,8,9,10]. 

Polybutyl methacrylate (PBMA) has broad application prospects as damping materials due to the large number of side groups, which can effectively increase internal friction. The glass transition temperature of PBMA is close to normal temperature [11,12,13,14], which also makes it more widely used. However, the peak value of internal friction of single homopolymer is low and the effective damping temperature range is narrow [8,10]. It is an effective way to widen the damping temperature range of polymer materials by adding small organic molecules and oligomer [15,16,17,18,19]. Jiyu Liang [20] investigated the effects of aliphatic resin (C5) content on the damping behavior of BIIR/BR vulcanizates. The tanδ peak shifted to higher temperature and the effective damping temperature range was broadened significantly with the increment content of C5 resin. Meng Song [18] studied the damping properties of the hindered phenol (AO-60)/nitrile-butadiene rubber (AO-60/NBR) composites. The addition of AO-60 had a positive impact on the damping of NBR due to the strong H-bonds between AO-60 and NBR matrix. AO-60/NBR had the best damping performance when the content of AO-60 was 36 phr. Wu [21] investigated the chlorinated butyl rubber/petroleum resins blends, which only showed a single tanδ peak. The position of tanδ peak was related to the softening point and the content of the petroleum resin. The loss peak increased and the peak location shifted to higher temperature with increasing the content of petroleum resins.

Petroleum resin C9 is a kind of aromatic oligomer derived from petroleum derivatives with the molecular weights range from 300 to 3000. C9 can significantly change the damping properties of polymer, which the promotion effect depends on the compatibility with the polymer matrix. Fengshun Zhang [22] investigated the effect of different petroleum resin on damping mechanism. The experimental results showed that the aliphatic C5 resin exhibited a better miscibility with chlorinated butyl rubber, compared with aromatic C9 resin. With increase in the content of the C5 resin, the effective damping temperature range was broadened remarkably. The damping performance of chlorinated butyl rubber was not improved by adding C9 due to poor compatibility. The damping properties in blends of poly (styrene-b-isoprene-b-styrene) (SIS) and hydrogenated aromatic hydrocarbon (C9) resin were investigated by Wu [23]. The addition of hydrogenated C9 resin had a positive impact on the damping of SIS due to the good compatibility between C9 and SIS. With the increasing softening point and content of the resin, the main tanδ peak shifted to higher temperatures and the useful damping temperature range was broadened.

In this work, PBMA and St were firstly copolymerized to improve the compatibility of matrix and filler. Then aromatic petroleum resin C9 was added to obtain the P(BMA-co-St)/C9 blends. The variation of damping property of PBMA with the content of C9 was investigated, aiming at preparing a damping material with wide damping temperature domain and high damping property.

## 2. Experimental Section

### 2.1. Materials

The butyl methacrylate (BMA), styrene (St), sodium persulfate, aluminium oxide, sodium dodecyl benzene sulfonate (SDBS), absolute ethyl alcohol, tetrahydrofuran, ethyl acetate were supplied by ChinaKelong Chemical Reagent Factory (Chengdu, China). The above chemicals were of analytical grade. Petroleum resin C5 and C9 was obtained from Shenzhen jitian chemical co. LTD (Shenzhen, China). All materials were used as received without further purification.

### 2.2. Preparation of P(BMA-co-St).

4 g of SDBS and 160 mL deionized water were added to a four-port flask with a mixer, a condensing tube, and the thermometer. BMA and St were added into a four-port flask for pre-emulsification at a temperature of 50 °C and the stirring speed of 400 r/min. After the pre-emulsification, 0.3 g sodium persulfate dissolved in 40 mL deionized water was added to the four-port flask drop by drop for 25 min under 75 °C. When the temperature of the system suddenly increased and the liquid level appeared blue, the remaining monomers were added immediately. The polymer emulsion was obtained after the reaction continued for 2.5 h to 3 h. Absolute ethyl alcohol was used to demulsification to obtain the P(BMA-co-St). The P(BMA-co-St) was washed and dried at 50 °C. Finally, the copolymer was pressed into splines (length of 20 mm, width of 12 mm and thickness of 3 mm) at the temperature of 160 °C and the pressure of 10MPa and the time of 20 min.

### 2.3. Preparation of P(BMA-co-St)/Petroleum Resin Blends

Petroleum resin with different mass ratio and 3 g of P(BMA-co-St) were added to a beaker containing 50 mL ethyl acetate solution and stirred at room temperature for 8 h to form an evenly solution. The mass ratio of C9 resin was 10%, 20% 30% 50%. The P(BMA-co-St)/petroleum resin blend was precipitated by adding absolute ethyl alcohol and deionized water and dried at 50 °C. Finally, the P(BMA-co-St)/petroleum resin blends were pressed into splines (length of 20 mm, width of 12 mm and thickness of 3 mm) at the temperature of 180 °C and the pressure of 10MPa and the time of 20 min.

### 2.4. Characterization

FTIR analysis of P(BMA-co-St) was carried out by the Thermo Fisher Scientific Nicolet iS50 infrared spectrometer (Waltham, MA, USA) with the scanning speed of 4 cm^−1^/min and the test range of 500 cm^−1^–4000 cm^−1^. Saturated polymer solution was prepared with tetrahydrofuran as solvent. KBr pellets were used in the test.

Hydrogen nuclear magnetic resonance (^1^HNMR) of P(BMA-co-St) was characterized by AV300 nuclear magnetic resonance spectrometer (Bruker, Germany). Test at room temperature with Tetramethylsilane (TMS) as internal standard.

The thermal properties of the P(BMA-co-St)/C9 blends were characterized by Mettler-Toledo DSC-822e. The sample mass was between 5 and 10 mg. The temperature was set as follows: firstly, it rose to 140 °C at the heating rate of 15 °C /min, then decreased to −10 °C to maintain for 3 min at the heating rate of 15 °C /min, and finally rose to 140 °C at the heating rate of 10 °C /min to record the data.

ZEISS EV0 MA15 scanning electron microscope was used to characterize the microstructure of the P(BMA-co-St)/C9 blends. The samples were fractured at low temperature and tested after treatment with gold spray.

Dynamic mechanical analysis (DMA) was carried out on a TA Q800 apparatus (TA Instruments, New Castle, DE, USA). The type of clamp was dual cantilever clamp. The testing method was temperature ramp-frequency sweep. During the test, the heating rate was 3 °C /min, the amplitude was 15 am, and the frequency was 1 Hz. The size of the sample was 20 mm long, 12 mm wide and 3 mm thick.

## 3. Results and Discussion

### 3.1. The Structure of P(BMA-co-St)

**Figure 1:**
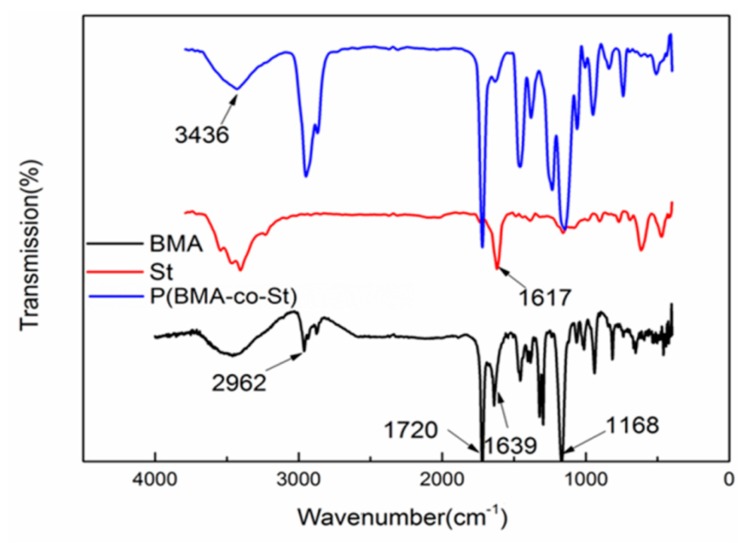
FTIR spectra of P(BMA-co-1wt% St).

FTIR has proven to be a powerful tool for analyzing the structure of copolymer. The FTIR spectra of P(BMA-co-St) is shown in Figure 1, the absorption peaks of 3000–2850 cm^−1^ were assigned to −CH_3_ and –CH_2._ The peaks at 1720 cm^−1^, 1639 cm^−1^ and 1168 cm^−1^ can be attribute to –C=O, C=C, –C–O–C–, respectively. In the curve of St, the peak of 1617 cm^−1^ can be attribute to –C=C– [24,25]. In the curve of P(BMA-co-St), the peak of –C=C– was weakened obviously. The peak of –C=O did not change either in P(BMA-co-1St) and BMA monomer. It is indicated that the copolymerization of polybutyl methacrylate and styrene could be seen initially.

**Figure 2:**
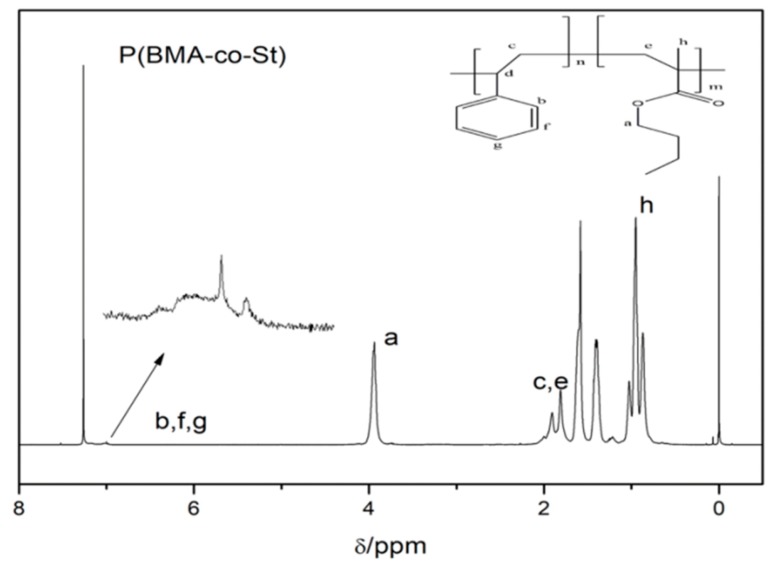
^1^HNMR spectra of P(BMA-co-1wt% St).

The composition of P (BMA-co-St) was determined using the ^1^H-NMR. Figure 2 and Table 1 show the chemical displacements of hydrogens of P(BMA-co-St). The characteristic chemical shifts of the hydrogen of the benzene ring (b, f, and g) are located at about 6.80 to 7.20 ppm. The signal shown at 1.75 to 2.00 belongs to –CH_2_– (c) and the peak at about 0.5 to 1.0 ppm belongs to –CH_3_ (h). The chemical shift peaks at about 3.80 to 4.10 ppm correspond to –OCH_2_– (a) [24,26]. Combined with the analysis of FTIR, it is known that P(BMA-co-St) was synthesized by emulsion polymerization.

### 3.2. Selection of the P(BMA-co-St) with the Most Suitable Content of St

**Figure 3:**
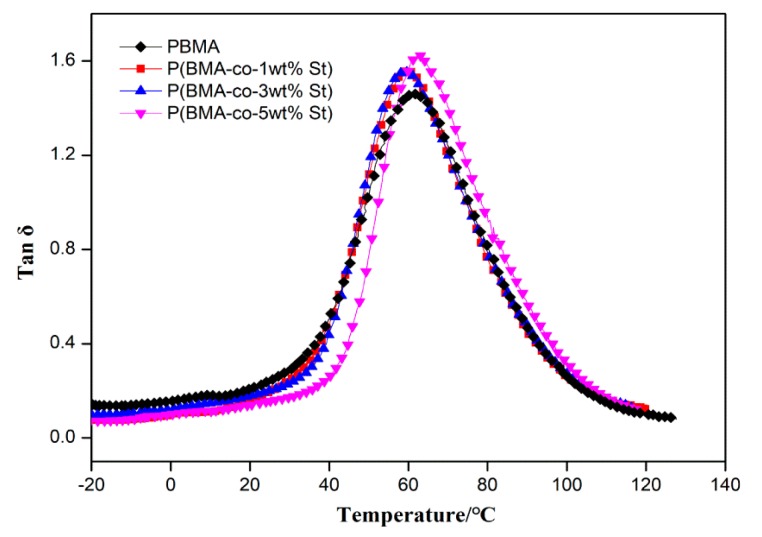
Temperature dependence of the loss tangent (tanδ) values for P(BMA-co-nSt).

In general, the addition of the second monomer affects the damping properties of the first monomer. In order to improve the compatibility of PBMA and C9 without affecting the damping properties of PBMA, the damping performance of P(BMA-co-nSt) copolymers were studied (n is the content of St, and the values of n are 0, 1 wt%, 3 wt% and 5 wt%). As shown in Figure 3 and Table S1, the glass transition temperature of PBMA did not change significantly after adding a small amount of St. The tanδ peak value of P(BMA-co-nSt) increased with the content of St, but the effective damping temperature range gradually narrowed. When the content of St was 5 wt% the effective damping temperature range was almost 7 °C lower than that of PBMA. Meanwhile, the composites became brittle after curing when the content of St is too much, which greatly affects the properties of PBMA and is not suitable for practical application. The damping properties of P(BMA-co-1wt%St) and pure PBMA are almost consistent. Therefore, P(BMA-co-1wt%St) was selected for further study.

### 3.3. Study on Compatibility between C9 and P(BMA-co-St)

**Figure 4:**
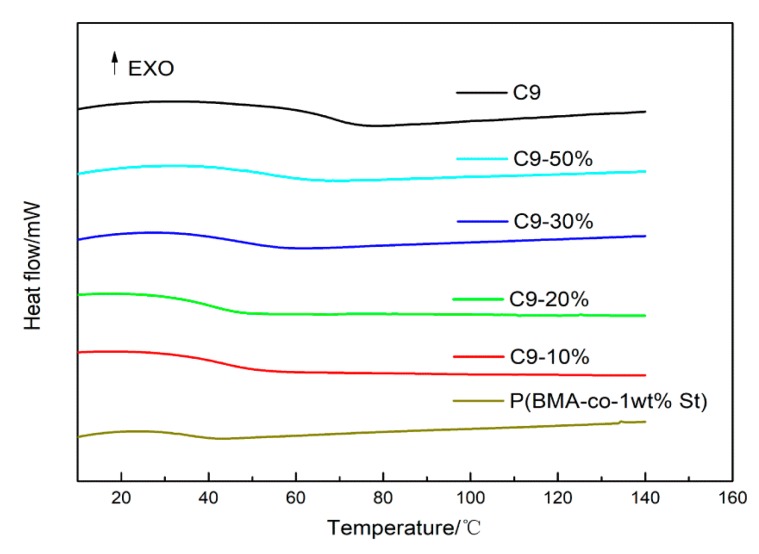
DSC curves of P(BMA-co-1wt%St)/C9 composites.

Figure 4 shows the DSC curves of P(BMA-co-1wt%St)/C9 composites with different mass ratio of C9. All curves express only one *Tg*, indicating the good compatibility between C9 and P(BMA-co-St). In addition, the *Tg* of P(BMA-co-1wt%St)/C9 composites gradually shift to high temperature with the increment content of C9. Microscopically, the change of *Tg* corresponds to the mobility of polymer segments. The *Tg* movement of P(BMA-co-1wt%St)/C9 composites towards high temperature is mainly due to the well dispersion of C9 resin in the P(BMA-co-1wt%St) matrix, which limited the movement of chain.

**Figure 5:**
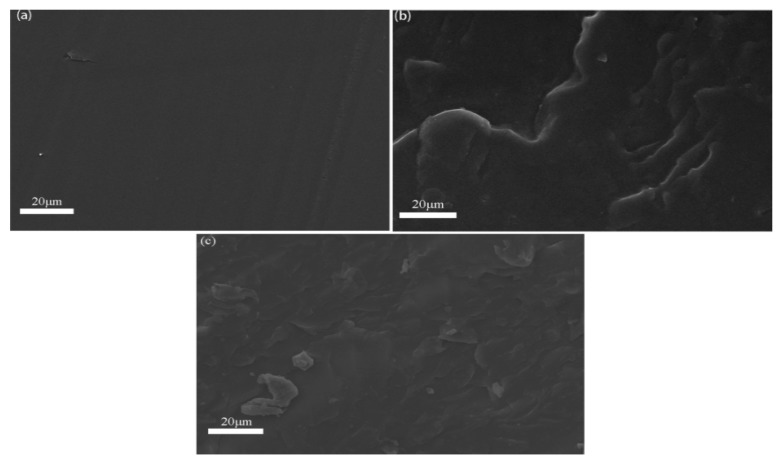
SEM images of (**a**) PBMA, (**b**) PBMA/C9, (**c**) P(BMA-co-1%St)/C9.

SEM was used to characterize the microstructure of the P(BMA-co-St)/C9 blends. As shown in Figure 5b, the PBMA/C9 showed an irregular fracture surface in the whole area. In Figure 5c, the fracture surface of P(BMA-co-1%St)/C9 is smoother than that of PBMA/C9, and there is no obvious phase separation. It can be explained that both C9 resin and St in P(BMA-co-St) contain benzene rings, so that P(BMA-co-St) have a good compatibility with C9.

**Figure 6:**
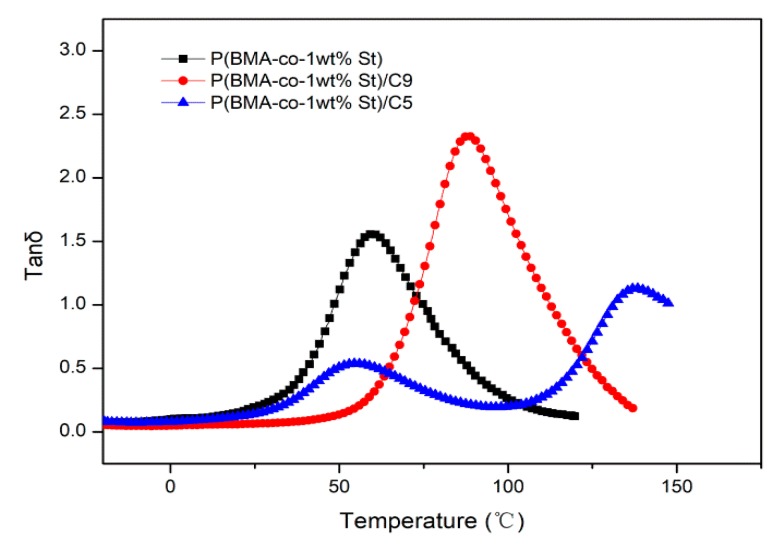
Temperature dependence spectra of tanδ of P(BMA-co-1wt%St)/C5 and P(BMA-co-1wt%St)/C9.

The compatibility between C9 and P(BMA-co-St) can also be explored by comparing the influence of aliphatic C5 resin without benzene ring and aromatic C9 resin on the damping performance of P(BMA-co-St), especially the variation of tanδ. From Figure 6, for P(BMA-co-1wt%St)/C5 composite, there are two tanδ peaks and the tanδ peak values are lower than that of P(BMA-co-1wt%St), indicated the poor compatibility between C5 and P(BMA-co-St). It can be seen that the tanδ peak value of P(BMA-co-1%St)/C9 composite is 2.33, which is higher than that of pure copolymers, and the tanδ peak also shifted significantly to higher temperature. It indicates that aromatic C9 resin has good compatibility with P(BMA-co-St).

**Figure 7:**
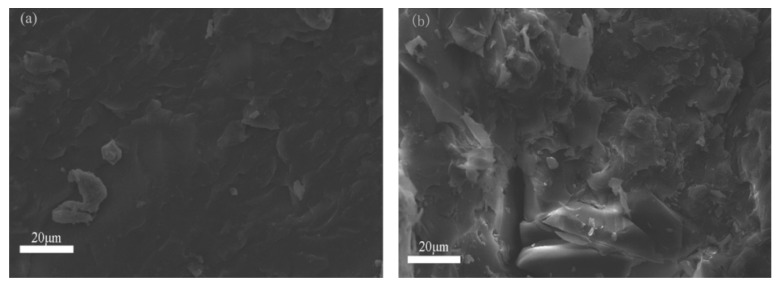
SEM images of (**a**) P(BMA-co-1%St)/C9, (**b**) P(BMA-co-1%St)/C5.

Figure 7 shows the SEM images of P(BMA-co-1wt%St)/C9 composite and P(BMA-co-1wt%St)/C5 composite. It can be seen that there is no obvious phase separation between C9 and matrix, while there is a biphase between C5 and matrix. The reason is that there is no benzene ring in the structure of C5, so it has poor compatibility with matrix. All of the above conclusions indicate that C9 with benzene ring in the structure has good compatibility with the matrix after the introduction of St copolymerization with PBMA.

### 3.4. Damping Properties of P(BMA-co-1wt%St)/C9 Composites

**Figure 8:**
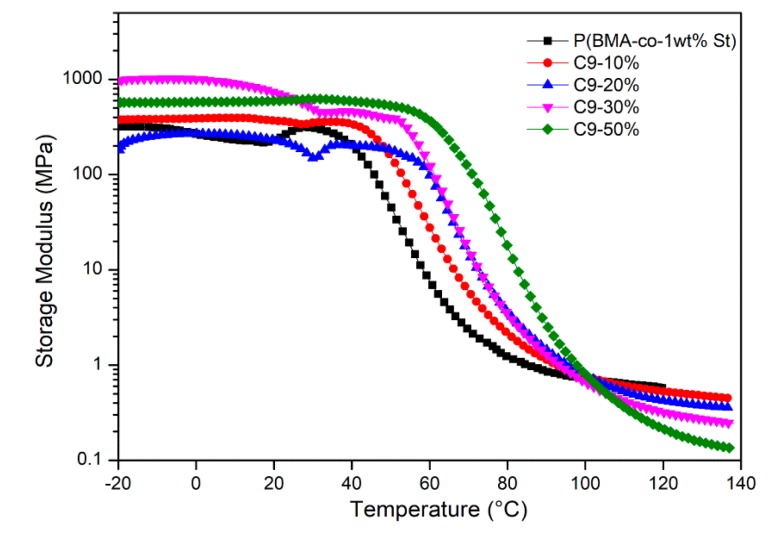
Temperature dependence of storage modulus of P(BMA-co-1wt%St)/C9 composites.

**Figure 9:**
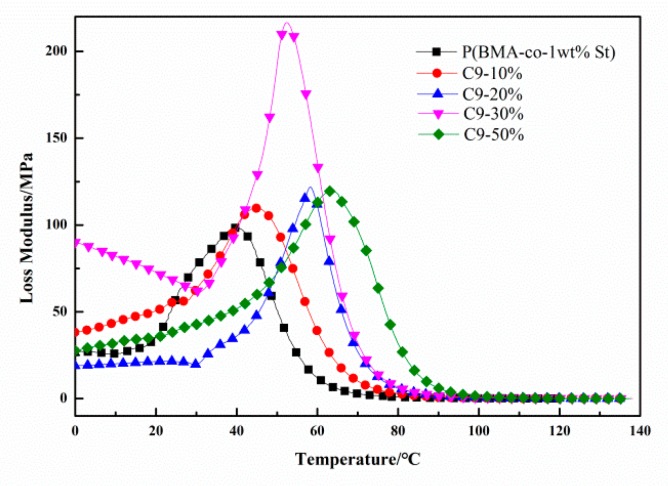
Temperature dependence of loss modulus of P(BMA-co-1wt%St)/C9 composites.

Dynamic mechanical analysis (DMA) was used to characterize the damping properties of P(BMA-co-1wt%St)/C9 composites. It can be seen from Figure 8 that the glass transition region move towards high temperature with the increment content of C9, which indicated that *Tg* of P(BMA-co-1wt%St)/C9 composites gradually increased. The storage modulus of P(BMA-co-1wt%St)/C9 decrease with the content of C9 at the high temperature. The reason is that C9 acted as plasticizer. The intermolecular interaction is reduced due to the short chains of C9 infiltrate into the long chain gap of P(BMA-co-1wt%St), resulting in the motion of larger-scale moving units (sub-rouse or rouse) [27], which greatly reduced the entanglement density of polymer chains and the modulus of rubber state. There is a trend of glass transition in Figure 9 that is the same as that of reflected in the DSC curve. All the curves showed only one glass transition temperature and increased with the content of C9.

**Figure 10:**
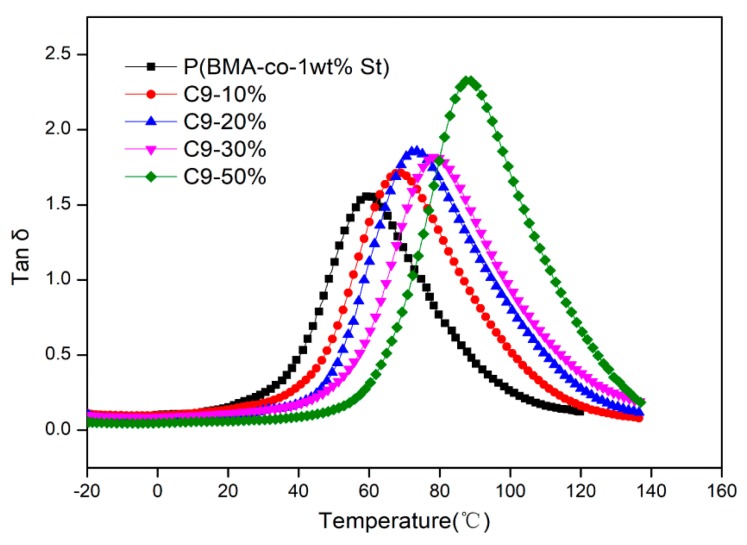
Temperature dependence of tanδ of P(BMA-co-1wt%St)/C9 composites.

Figure 10 shows the temperature dependence curves of tanδ of P(BMA-co-1wt%St)/C9 composites. The data are also recorded in Table S2. It can be seen that the tanδ peak value of P(BMA-co-1wt%St) is 1.56 and corresponding damping temperature range is 66.47 °C. There are many benzene rings in C9 resin, and the St in P(BMA-co-1wt%St) also contains benzene ring, which made the good compatibility between C9 resin and P(BMA-co-1wt%St). For P(BMA-co-1wt%St)/C9 composites, the tanδ peak value increase with the content of C9. The glass transition temperature gradually moved to high temperature, which is consistent with the results of DSC. The tanδ peak value of (BMA-co-1wt%St)/C9-10% is 1.71 and the corresponding damping temperature range is 69.24 °C. The tanδ peak value of P(BMA-co-1wt%St)/C9-50% is 2.33 and the corresponding damping temperature range is 71.74 °C. It indicates that C9 effectively improved the damping properties of P(BMA-co-1wt%St).

**Figure 11:**
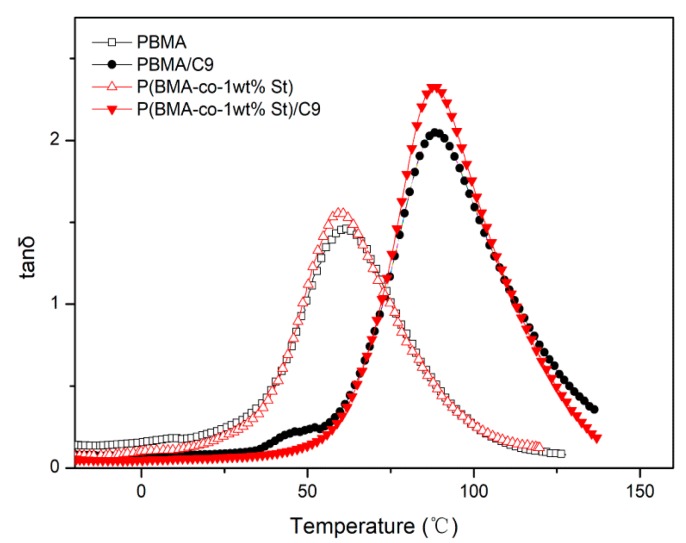
Temperature dependence of tanδ of P(BMA-co-1wt%St)/C9 composites.

**Figure 12:**
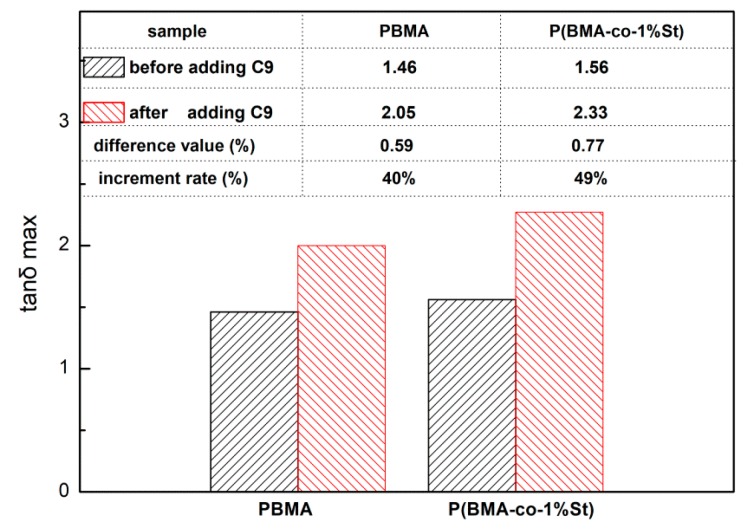
The tanδ_max_ of P(BMA-co-nSt)/C9 composites.

As shown in Figure 11, the tanδ peak of P(BMA-co-1wt%St)/C9 is higher than that of PBMA, PBMA/C9 and P(BMA-co-1wt%St). The tanδ peak value of PBMA and P(BMA-co-1wt%St) are 1.46, 1.56, respectively. The tanδ peak value of PBMA/C9, and P(BMA-co-1wt%St)/C9 are 2.05, 2.33, respectively. The increment rate of tanδ_max_ was calculated to show the improvement of damping properties of PBMA. As shown in Figure 12, the increment rate of tanδ_max_ of P(BMA-co-1wt%St)/C9 and P(BMA-co-5wt%St)/C9 are 49% and 45% respectively, which are much higher than PBMA/C9. It indicated that the introduction of St greatly promoted the compatibility between C9 and PBMA. According to the references, many works, such as inorganic particles [11] and interpenetrating network [13], had positive effects on the damping properties of PBMA. Compared with their results, the tanδ_max_ of P(BMA-co-5wt%St)/C9 was as high as or even higher than that of them, indicating that the damping properties of PBMA can be effectively improved by adding C9.

## 4. Conclusions

The effects of C9 resin loading on damping behavior of PBMA have been investigated. A trace amount of St was introduced to copolymerized with PBMA to improve the compatibility between C9 and PBMA without changing the properties of PBMA bulk. The tanδ peak shifted to high temperature obviously and the effective damping temperature range was widened remarkably with the increment content of C9. The influence of aromatic resin C9 and aliphatic resin C5 on PBMA damping performance was compared. There was a good compatibility between C9 and PBMA, and the effect of C9 on the damping performance of PBMA was much better than that of C5. Therefore, the P(BMA-co-St)/C9 could be considered as be a competent candidate for damping materials with wide damping temperature domain and high damping property.

## Figures and Tables

**Table 1:** Chemical displacements of different hydrogen of P(BMA-co-1wt%St).

Chemical Displacements/ppm	Proton Type
1.00~0.50	–CH_3_ (h)
2.00~1.75	–CH_2_– (c,e)
4.10~3.80	–OCH_2_– (a)
6.80~7.20	=CH– (b,f,g)

## Supplementary Materials

The following are available online at https://www.mdpi.com/2073-4360/12/3/543/s1, Table S1: The damping properties data of P(BMA-co-nSt), Table S2: The damping properties data of P(BMA co 1wt% St)St)/C9.
