# Supplementary material for: Effect of Aromatic Petroleum Resin on Damping Properties of Polybutyl Methacrylate
Source: Polymers (Basel). 2020 Mar 2;12(3):543. doi: 10.3390/polym12030543 (PMC7182884; doi:10.3390/polym12030543)
Supplement: Supplementary file 1 [file polymers-12-00543-s001.pdf]

polymers

Supporting Information

Effect of Aromatic Petroleum Resin on Damping Properties of Polybutyl Methacrylate

Songhan Wan [1], Saisai Zhou [1], Xing Huang [1], Songbo Chen [1], Shuwei Cai [1], Xianru He [1,*] and Rui Zhang [2]

[1] School of Materials Science and Engineering, Southwest Petroleum University, Chengdu, 610500, China; songhanwan@foxmail.com (S.W.); zhousaisai@xinpoint.com (S.Z.); huangxing0226@foxmail.com (X.H.); chen756424838@foxmail.com (Songbo Chen); victor8951@foxmail.com (Shuwei Cai)
[2] Institute für Physik, Universität Rostock, Albert-Einstein-Str. 23-24, 18051 Rostock, Germany; rui.zhang@uni-rosotck.de

* Correspondence: xrhe@swpu.edu.cn

Table S1. The damping properties data of P(BMA-co-nSt).

Sample	Tanδ Max		Temperature Range of tanδ > 0.3			TA (tanδ > 0.3)
P(BMA-co-nSt)	Value	T/°C	T1/°C	T2/°C	ΔT/°C	
0%	1.46 ± 0.02	60.8 ± 0.1	31.3 ± 0.3	97.8 ± 0.2	66.5 ± 0.5	37.2 ± 0.2
1%	1.56 ± 0.04	60.4 ± 0.3	32.6 ± 0.4	98.4 ± 0.2	65.8 ± 0.6	37.7 ± 0.2
3%	1.56 ± 0.01	59.6 ± 0.2	35.9 ± 0.3	98.5 ± 0.3	62.6 ± 0.6	38.0 ± 0.1
5%	1.73 ± 0.03	62.5 ± 0.1	41.76 ± 0.2	101.7 ± 0.3	59.89 ± 0.5	37.75 ± 0.2

Table S2. The damping properties data of P(BMA-co-1wt%St)/C9.

Sample Code	Tanδ Max		Temperature Range of tanδ > 0.3			TA (tanδ > 0.3)
P(BMA-co-1wt%St)/C9	Value	T/°C	T1/°C	T2/°C	ΔT/°C	
0%	1.56 ± 0.04	60.4 ± 0.3	32.6 ± 0.4	98.4 ± 0.2	65.8 ±0.6	37.7 ± 0.2
10%	1.71 ± 0.03	68.3 ± 0.2	40.4 ± 0.2	109.8 ± 0.4	69.43 ± 0.6	46.5 ± 0.4
20%	1.86 ± 0.04	73.09 ± 0.2	47.0 ± 0.4	118.5 ± 0.3	71.53 ± 0.7	54.0 ± 0.3
30%	1.82 ± 0.02	78.28 ± 0.3	50.0 ± 0.2	125.2 ± 0.4	75.23 ± 0.6	51.3 ± 0.5
50%	2.33 ± 0.03	88.11 ± 0.4	59.3 ± 0.5	132.3 ± 0.3	73.04 ± 0.8	67.3 ± 0.4
